# Gastrointestinal Bleeding Risk with Oral Anticoagulant and Proton Pump Inhibitor Co-Therapy in Nursing Homes

**DOI:** 10.1093/geroni/igaf122.3386

**Published:** 2025-12-31

**Authors:** Iju Shakya, Yoojin Lee, Andrew Zullo, Jimmie Roberts, Sarah Berry

**Affiliations:** Brown University, Providence, Rhode Island, United States; Brown University, Providence, Rhode Island, United States; Brown University, Providence, Rhode Island, United States; Hebrew SeniorLife, Boston, Massachusetts, United States; Hebrew SeniorLife & Harvard Medical School, Hebrew SeniorLife, Massachusetts, United States

## Abstract

Direct oral anticoagulants (DOACs) are essential for preventing and treating thrombosis but increase upper gastrointestinal bleeding (UGIB) risk. Proton pump inhibitors (PPIs) may reduce UGIB risk, but it is unclear whether this protective effect extends to nursing home (NH) residents, who are at high bleeding risk. We aimed to estimate the effect of DOAC and PPI co-therapy, compared to DOAC only, on 1-year UGIB hospitalization risk among NH residents. We conducted a retrospective cohort study emulating a hypothetical target trial. Adults aged ≥ 66 years, enrolled in fee-for-service Medicare, and living in NHs between March 1, 2013, and December 31, 2019, were included. Residents were new DOAC users (no use in prior 60 days) and were classified as exposed if they initiated PPI within 31 days of DOAC dispensing or as unexposed if they did not. UGIB hospitalizations were identified from claims during one-year follow up. A clone-censor-weight approach addressed immortal time bias and discrete-time hazards models estimated UGIB risk in each treatment group. Among 149,686 new DOAC episodes, 10,445 (7.0%) were exposed to PPI co-therapy while 139,241 (93.0%) were unexposed. UGIB incidence was similar in the exposed group (2.8%) and the unexposed group (2.5%). Further analysis with covariate adjustment is needed, but our preliminary results do not suggest a difference in UGIB with DOAC-PPI co-therapy in NH residents.

